# Actinomycosis of the middle turbinate

**DOI:** 10.1002/ccr3.6289

**Published:** 2022-08-26

**Authors:** Malek Mnejja, Imen Achour, Asma Abbes, Marwa Regaieg, Walid Bouayed, Rachid Jlidi, Bouthaïna Hammami, Ilhem Charfeddine

**Affiliations:** ^1^ ENT Department Habib Bourguiba Hospital Faculty of Medicine ‐ University of Sfax Sfax Tunisia; ^2^ Private Practice Sfax Tunisia

**Keywords:** actinomycosis, middle turbinate

## Abstract

Actinomycosis is an uncommon bacterial disease caused by actinomyces. Cervicofacial infection accounts for more than 60% of all cases. However, nasal and paranasal sinus involvement has rarely been described. We report herein a case of a patient presenting with middle turbinate actinomycosis.

## Abstract 2

The diagnosis of nasal and paranasal actinomycosis should be kept in mind even though it is a rare condition. It has no specific clinic presentation nor pathognomonic scanographic features. The confirmation of the diagnosis is microbiological. Histology examination is useful. Its treatment is Surgical and medical, combined. A minimum of two to six weeks of general antibiotic therapy is needed. Follow‐up care is essential.

## 1 INTRODUCTION

Actinomycosis is an uncommon bacterial disease caused by Actinomyces, gram‐positive anaerobes. Most cases are odontogenic and predominantly occur in immunocompetent individuals.^1^ Four major clinical forms of actinomycosis exist in humans: cervicofacial, thoracic, abdominopelvic, and central nervous system (CNS).^2^ Cervicofacial infection accounts for more than 60% of all cases.^1^ However, nose and paranasal sinus involvement has rarely been reported.^3^, ^4^, ^5^, ^6^, ^7^, ^8^, ^9^, ^10^ We describe herein a case report of a patient presenting with middle turbinate actinomycosis. It is the second case reported in the literature.

## 2 CASE REPORT

**FIGURE 1:**
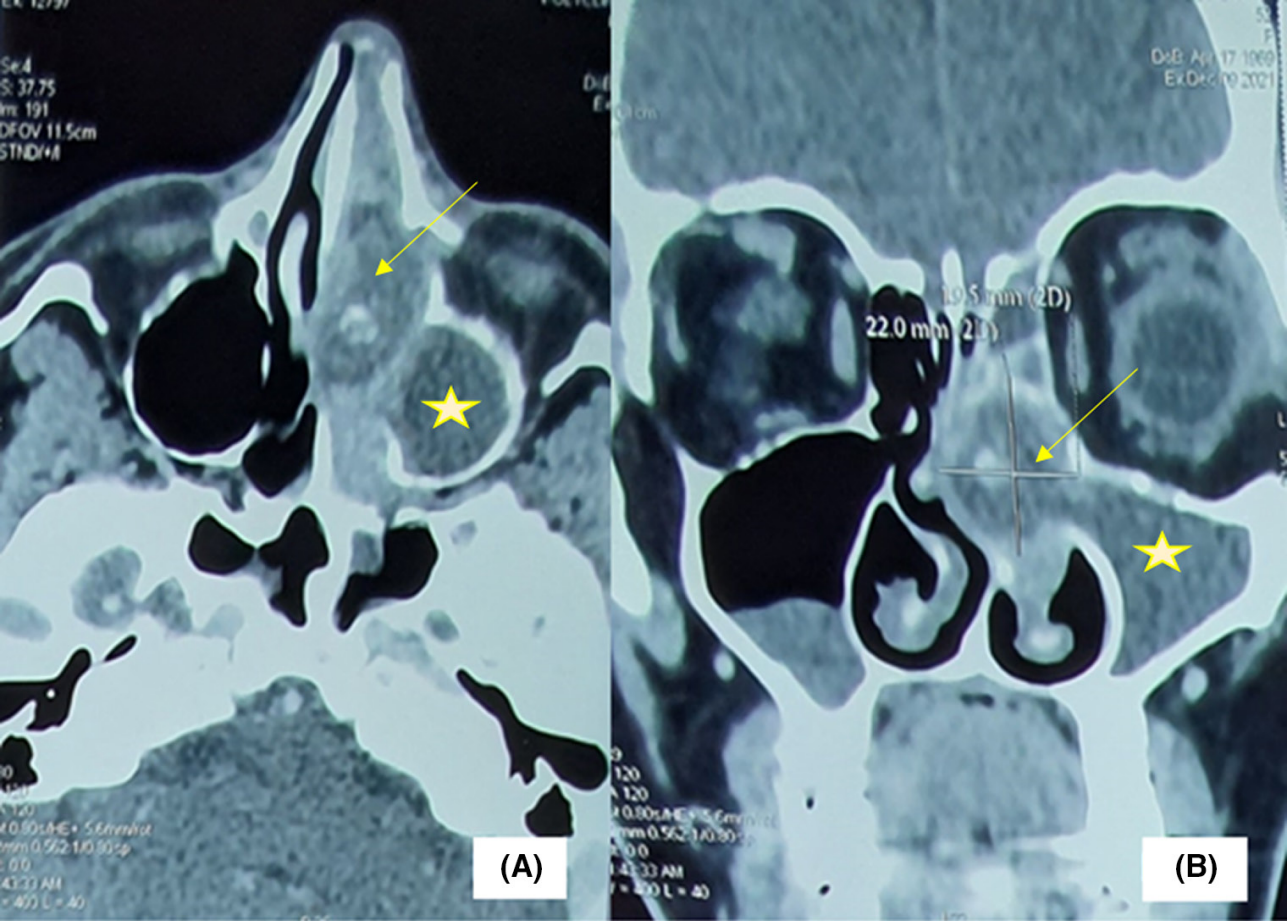
Axial (A) and coronal (B) paranasal sinus computed tomography scan showing a heterogeneous left‐sided nasal mass attached to the medial turbinate (arrow) with an ipsilateral maxillary and ethmoid sinuses opacities (stars)

**FIGURE 2:**
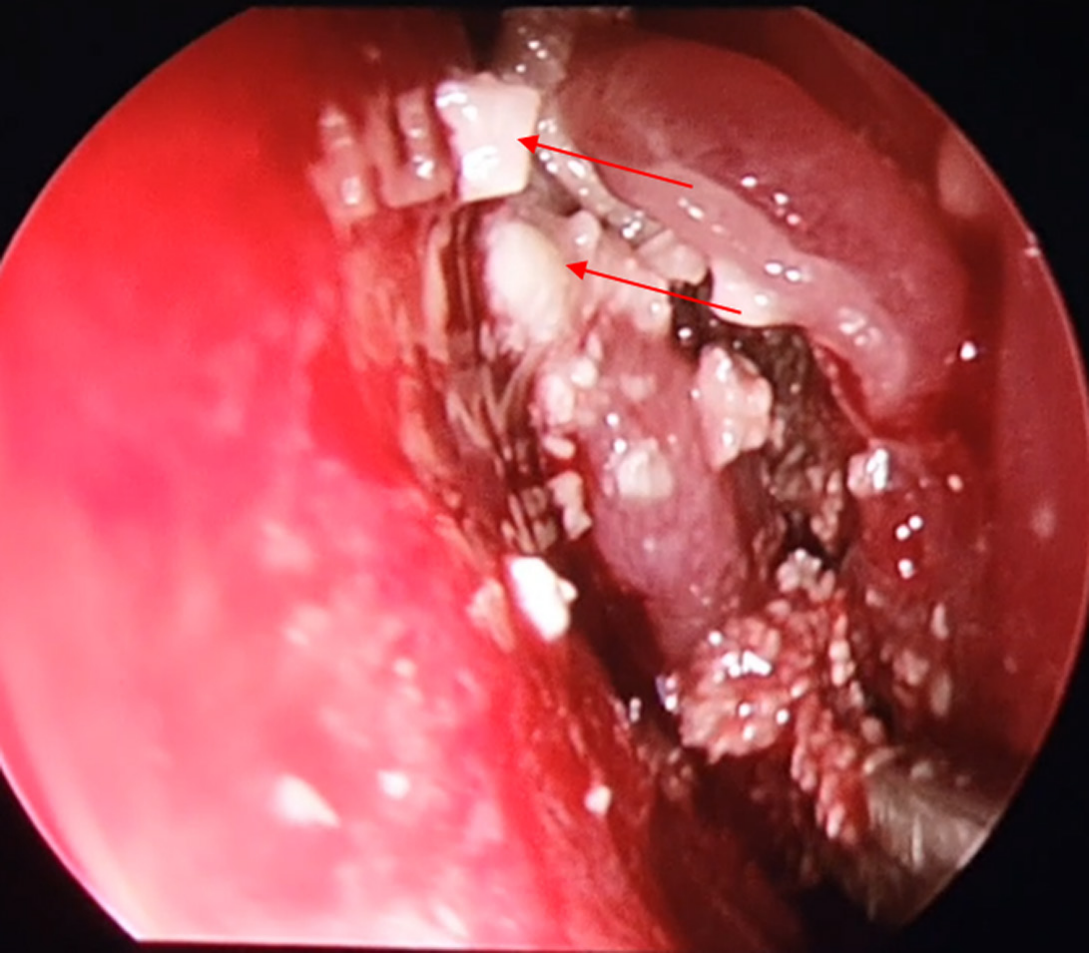
Intraoperative endoscopic imaging showing characteristic white lumps (red arrows)

**FIGURE 3:**
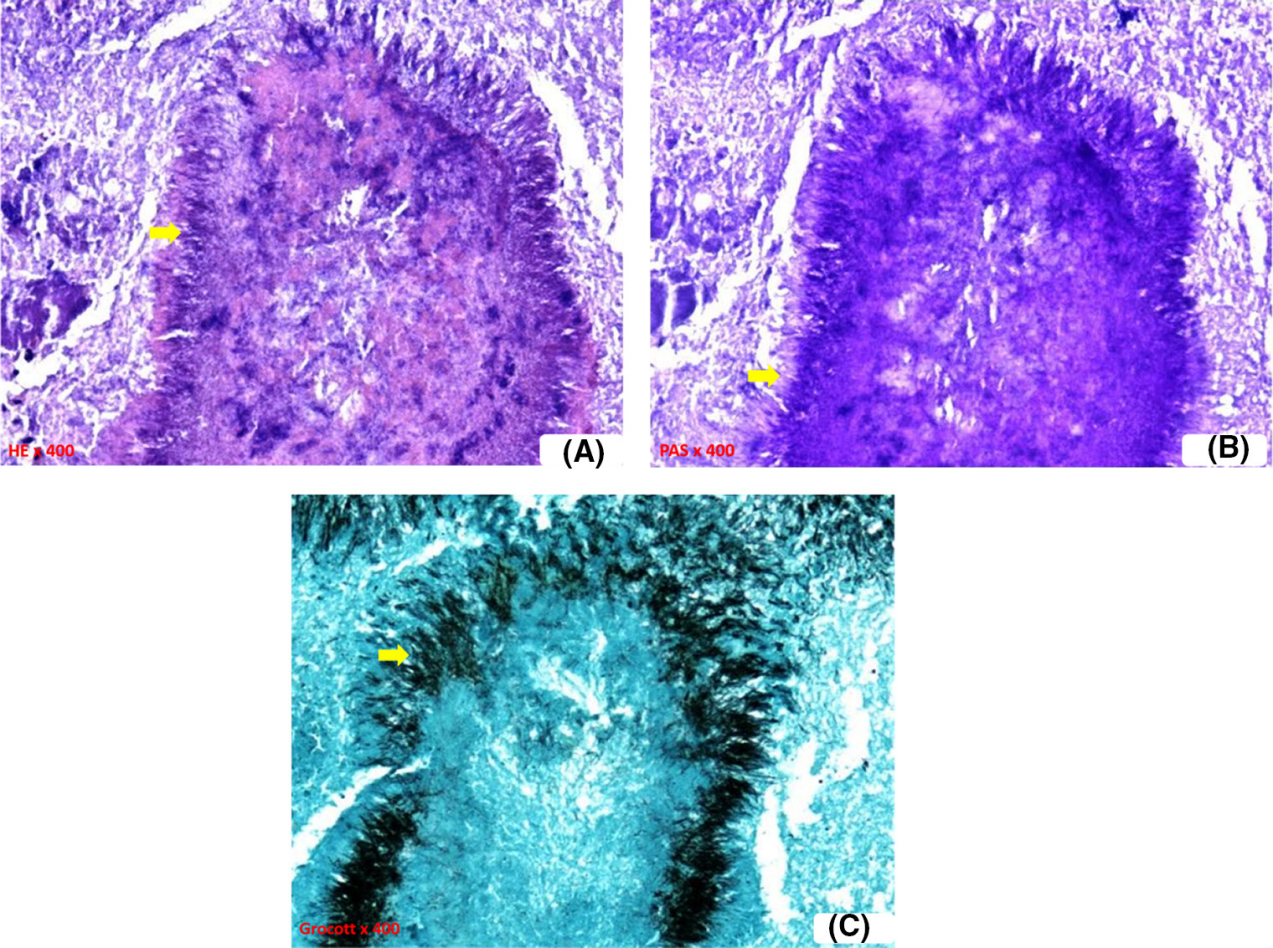
Branching filaments (arrows) with peripheral fibrinous and leukocytic exudate adjacent to suppurative infiltrates after Hematoxylin and eosin (A), Periodic acid–Schiff (B) and Grocott staining (C), respectively

A 55‐year‐old female patient was referred to our outpatient clinic for a 3‐month history of a left nasal obstruction concomitant with purulent nasal discharge and facial algia nonresponding to many courses of oral antibiotics. Her medical history included diabetes mellitus. The patient did not relate any dental history nor facial trauma history. The endoscopic examination revealed a purulent rhinorrhoea and a hypertrophic middle turbinate with granulomatous mucosa, filling the nasal cavity and repressing the septum. Oral cavity examination did not reveal any dental abnormality. Computed tomography scan of the paranasal sinuses showed a heterogeneous lesion of the left Middle turbinate focally hyperdense filling the nasal cavity and repressing the septum. Ipsilateral maxillary, ethmoid, and frontal sinuses were entirely filled. No sinus wall erosion was noted (Figure 1). Fungal sinusitis was suspected. Our patient underwent a functional endoscopic sinus surgery consisting of a left middle turbinoplasty, a left middle meatotomy, and a left functional endoscopic ethmoidectomy. A sphenoidotomy was also performed since the lesion was intraoperatively extensive to the sphenoid ostium. However, the presence of white lumps, intraoperatively, was in favor of actinomycosis (Figure 2). Histopathology confirmed indeed the latter diagnosis given the presence of actinomycetes (Figure 3). Thus, the patient received a 4‐week‐oral amoxicillin‐clavulanic acid cure (80 mg/kg/day). The clinic and endoscopic 6‐month follow‐up did not reveal any sign of relapse.

## 3 DISCUSSION

More than half of the reported cases of actinomycosis have a cervicofacial localization. However, nasal and paranasal sinus involvement has rarely been described.^11^ Only one case of middle turbinate actinomycosis was reported in the English literature.^12^

Although the term actinomyces has a Greek origin meaning “ray fungus,” actinomycosis is a chronic bacterial granulomatous infection caused by gram‐positive, anaerobic to microaerophilic bacteria that are not acid‐fast.

*Actinomyces israelii* is the most common human pathogen of actinomycosis that inhabits oral and buccal cavities; it is considered to be an endogenous commensal organism.^13^ Hence, the loss of mucosal integrity by direct trauma, tooth extraction, root canal therapy, periodontal or periapical lesions are incriminated in the onset of the disease.^11^, ^14^ However, our patient did not have any dental history and buccal examination did not reveal any abnormalities.

Patients usually present with non‐specific unilateral nasal symptoms consistent with chronic sinusitis, such as purulent nasal discharge, nasal obstruction, foul odor, and sinusalgia.^15^, ^16^

Paranasal sinuses computed tomography imaging does not permit a specific diagnosis. It shows opacities in the paranasal sinus, focal calcified lesions, and/or focal areas of bone destruction. However, it allows a more accurate definition of the dimensions and extension of the infection.^1^ Given the imaging findings, many other differentials may be evoked as has been mentioned in our case report. Nasal and paranasal actinomycosis has to be differentiated from nocardiosis, fungal sinusitis, and neoplasms.^9^

Positive bacterial culture confirms the diagnosis. However, its low rate of isolation makes it difficult. No bacterial examination was carried out for our patient.

Histologic examination reveals the characteristic sulfuric granules in 30% of cases. They are described as tiny, yellow‐white, lobulated, grainy microcolonies with club‐shaped filaments, measuring 1–5 μm in diameter and radiating in a rosette pattern, surrounded by inflammatory cells. Our patient histologic findings were consistent (Figure 3). However, sulfuric granules are not pathognomonic since they have also been described in nocardiosis and botryomycosis.^9^, ^12^

As for the therapeutic recommendations, both surgical and medical treatments should be combined. Vascular supply decreases in actinomycosis‐infected tissues, making the penetration of antibiotics to the lesion difficult. Therefore, the lesion should be surgically removed and the surrounding tissues thoroughly debrided.^13^, ^17^ Then, surgery should be followed by a long‐term‐penicillin therapy; Penicillin G (50–75 mg/kg/day intravenously in four daily divided doses) for 4–6 weeks followed by peroral penicillin V (30–60 mg/kg/day administered in four divided doses) for 2–12 months.^1^

If the patient is known allergic to penicillin, tetracycline, clindamycin, cephalosporin, or erythromycin may be prescribed.^10^, ^12^, ^13^

Fluoroquinolones, aztreonam, fosfomycin, and other aminoglycosides are known to have poor activity against Actinomyces species.^2^, ^18^

However, no consensus has been reached on the antibiotic therapy duration. It has been established that patients with cervicofacial actinomycosis have a favorable prognosis since some occasional cures with aggressive surgery alone have been reported in the pre‐antibiotic era.^19^

The duration of the antibiotic therapy should be individualized based on the site of the infection, the clinical and the radiologic response to the treatment and its severity. Short courses‐regimen consisting of 2–6 weeks of oral antibiotic therapy (± intravenous) associated with surgical debridement have been reported to be curative in recent studies.^7^, ^10^, ^19^ A thorough and prolonged follow‐up is required to watch for recurrence which might happen after several years.^16^

As for our patient, endoscopic surgical treatment was followed by a 4‐week oral antibiotherapy (80 mg/kg/day of oral amoxicillin‐clavulanic acid). No signs of relapse were detected during her 6‐month follow‐up care.

## 4 CONCLUSION

Nasal and paranasal actinomycosis is a rare bacterial disease mimicking fungal and neoplastic pathologies. Its diagnosis is microbiological and/or histologic. The treatment is based on endoscopic surgery and antibiotics.

However, more studies are needed to standardize the minimum required duration of antibiotic therapy.

## AUTHOR CONTRIBUTIONS

Malek Mnejja, Walid Bouayed, Imen Achour, and Rachid Jlidi involved in patient care, data and information collection, manuscript preparation, and manuscript review. Asma Abbes and Marwa Regaieg involved in manuscript preparation. Bouthaïna Hammami and Ilhem Charfeddine involved in manuscript review.

## FUNDING INFORMATION

None.

## ACKNOWLEDGEMENTS

None.

## CONFLICT OF INTEREST

The authors declare no potential conflicts of interest.

## ETHICAL APPROVAL

No ethical conflicts to disclose.

## CONSENT

Written informed consent was obtained from the patient for the publication of this case report and accompanying clinical images.

## Data Availability

The datasets are available from the corresponding author on reasonable request.

## References

[1] Oostman O , Smego RA . Cervicofacial actinomycosis: diagnosis and management. Curr Infect Dis Rep. 2005;7(3):170‐174. 15847718 10.1007/s11908-005-0030-0

[2] Smego RA , Foglia G . Actinomycosis. Clin Infect Dis. 1998;26(6):1255‐1261. 9636842 10.1086/516337

[3] Wadhera R , Gulati SP , Garg A , Ghai A , Kumar S . Frontal sinus actinomycosis presenting as osteomyelitis of frontal bone. Otolaryngol Head Neck Surg. 2008;138(4):544‐545. 18359374 10.1016/j.otohns.2007.12.009

[4] Vorasubin N , Wu AW , Day C , Suh JD . Invasive sinonasal actinomycosis. Laryngoscope. 2013;123(2):334‐338. 23008010 10.1002/lary.23477

[5] Kim SD , Kim DS , Choi KU , Cho KS . Nasal cavity actinomycosis mimicking rhinolith. J Craniofac Surg. 2018;29(3):e255‐e257. 29461370 10.1097/SCS.0000000000004304

[6] Zalagh M , Akhaddar A , Benariba F . Chronic rhinorrhea revealing an actinomycotic rhinolithiasis with ectopic tooth. Int J Oral Maxillofac Surg. 2012;41(3):297‐299. 21865012 10.1016/j.ijom.2011.07.901

[7] Won HR , Park JH , Kim KS . Simultaneous actinomycosis with aspergillosis in maxillary sinus. Br J Oral Maxillofac Surg. 2013;51(4):e51‐e53. 22445647 10.1016/j.bjoms.2012.03.003

[8] Kim JS , Noh SJ , Ryu SH . Osteoma with actinomycosis in a nasal cavity. Med (United States). 2017;96(51):e9376. 10.1097/MD.0000000000009376 PMC5758238 29390536

[9] Batzakakis D , Karkos PD , Papouliakos S , Leong SC , Bardanis I . Nasal actinomycosis mimicking a foreign body. Ear Nose Throat J. 2013;92(7):E16. 10.1177/014556131309200715 23904310

[10] Numano Y , Nomura K , Watanabe M , et al. Paranasal sinus actinomycosis treated with a combination of surgery and long‐term low‐dose macrolide. Ear Nose Throat J. 2022;14556132210922. doi:10.1177/01455613221092208 35400239

[11] Nimmagadda SV , Man L‐X , McKenna MK , Faria JJ , Schmale IL . Actinomyces acute rhinosinusitis complicated by subperiosteal abscess in an immunocompromised 12‐year‐old: case report and literature review. Case Rep Otolaryngol. 2022;2022:1‐9. 10.1155/2022/7058653 PMC9015867 35444837

[12] Özcan C , Talas D , Görür K , Aydin Ö , Yildiz A . Actinomycosis of the middle turbinate: an unusual cause of nasal obstruction. Eur Arch Oto‐Rhino‐Laryngol. 2005;262(5):412‐415. 10.1007/s00405-004-0832-y 15549341

[13] Bennhoff DF . Actinomycosis: diagnostic and therapeutic considerations and a review of 32 cases. Laryngoscope. 1984;94(9):1198‐1217. 6381942 10.1288/00005537-198409000-00013

[14] Weese WC , Smith IM . A study of 57 cases of actinomycosis over a 36‐year period: a diagnostic “failure” with good prognosis after treatment. Arch Intern Med. 1975;135(12):1562‐1568. 1200725

[15] Woo HJ , Bae CH , Song SY , Choi YS , Kim YD . Actinomycosis of the paranasal sinus. Otolaryngol Head Neck Surg. 2008;139(3):460‐462. 18722231 10.1016/j.otohns.2008.06.001

[16] Park KS , Lee DH , Lim SC . Actinomycosis of the nasal cavity. Braz J Otorhinolaryngol. 2021. doi:10.1016/j.bjorl.2021.05.003 PMC9734254 34112606

[17] Weese WC , Ian M . A study of 57 cases of over a 36‐year period actinomycosis. 2015. 1200725

[18] Tanaka‐Bandoh K , Watanabe K , Kato N , Ueno K . Susceptibilities of actinomyces species and Propionibacterium propionicus to antimicrobial agents. Clin Infect Dis. 1997;25 Suppl 2:S262‐S263. 9310699 10.1086/516187

[19] Sudhakar SS , Ross JJ . Short‐term treatment of actinomycosis: two cases and a review. Clin Infect Dis. 2004;38(3):444‐447. 14727221 10.1086/381099

